# Brief Report: Understanding Program-Level Impact of COVID-19 in Lung Cancer Screening Programs in the United States

**DOI:** 10.1016/j.jtocrr.2024.100709

**Published:** 2024-07-31

**Authors:** Valeda Yong, Lynde Lutzow, Andrew Ciupek, Angela Criswell, Jennifer C. King, Grace X. Ma, Cherie P. Erkmen

**Affiliations:** a Department of Surgery, Temple University Hospital, Philadelphia, Pennsylvania; b GO2 for Lung Cancer, Washington, District of Columbia; c Center for Asian Health, Lewis Katz School of Medicine at Temple University, Philadelphia, Pennsylvania; d Department of Thoracic Medicine and Surgery, Lewis Katz School of Medicine at Temple University, Philadelphia, Pennsylvania

**Keywords:** Lung cancer screening, COVID-19 pandemic, Lung cancer, Early detection, Health care delivery

## Abstract

**Introduction:**

Lung cancer screening (LCS) reduces lung cancer mortality, yet uptake pre– and post–coronavirus disease 2019 (COVID-19) remains low. The impact of COVID-19 on LCS programs across the United States is unknown. Ours is the first multi-institutional study to identify barriers to LCS experienced during the pandemic. Our work will hopefully inform the development of targeted resources to facilitate increased uptake of LCS.

**Methods:**

A nationwide survey of Centers of Excellence (SCOE) in LCS was conducted by GO2 for Lung Cancer Foundation. In 2021, survey items included questions regarding program structure, screening rates, and systemic barriers to LCS delivery experienced amid COVID-19.

**Results:**

A total of 99 programs representing 1112 screening sites responded. A median of 868 patients were screened during the year of 2020. Patient recruitment, patient education, and in-person service access were negatively affected by COVID-19, whereas the use of telemedicine was positively affected. Coordination of care and timely reporting of results were largely unaffected by the pandemic.

**Conclusions:**

Our findings provide a real-world snapshot of how COVID-19 affected LCS from a program perspective. These findings highlight ongoing challenges with educating and engaging those at high risk for lung cancer in LCS. Program resources should be directed toward increasing adherence to LCS among eligible patients.

## Introduction

Despite recent advances in treatment and decreasing smoking rates, lung cancer continues to cause nearly as many cancer-related deaths for U.S. men and women as colon, breast, and prostate cancers combined.^1^ Lung cancer screening (LCS) through low-dose computed tomography (LDCT) decreases the risk of dying from lung cancer by 20% to 25%. Yet, utilization remains low,^2^^,^^3^ especially among populations experiencing health disparities.^4^ The unprecedented global pandemic of coronavirus disease 2019 (COVID-19) prompted the Centers of Medicare and Medicaid Services to recommend delaying non-essential procedures such as LCS.^5^ In addition, expert consensus recommended delay of LCS to reduce COVID-19 exposure for this vulnerable group of high-risk individuals and conserve hospital resources.^6^ Single-institution reports of commencing LCS resulted in fewer patients screened and increased rates of suspicious nodules on screening.^7^ LCS among populations experiencing health disparities remained low amid COVID-19.^4^^,^^8^ How LCS programs across the United States emerged from COVID-19 restrictions is poorly understood. Because low uptake of LCS presents a missed opportunity to save lives, program-level barriers, and facilitators of LCS uptake must be understood. Understanding the impact of COVID-19 on operational components of LCS programs can inform the allocation of resources to increase uptake in the future. To address this need, we surveyed a nationwide sample of LCS programs to better understand the impact of COVID-19 on their LCS program components.

## Materials and Methods

We partnered with GO2 for Lung Cancer (GO2), a nonprofit patient advocacy organization dedicated to improving lung cancer outcomes and survivorship, to conduct a qualitative study of LCS programs designated as Centers of Excellence in Lung Cancer Screening (SCOEs) by GO2 during the start of the COVID-19 pandemic.^9^ In addition to the annual survey of program geography, patient demographics, and LCS and diagnoses, questions regarding focus on populations experiencing health disparities and facilitators and barriers to LCS amid COVID-19 were added to the 2021 survey.

SCOEs were invited to participate through email. Descriptive statistical analysis was completed for aggregate program data collected in the Summer of 2021, reflecting the screening experience during the 2020 calendar year. The primary outcome of interest was understanding program components most significantly affected by COVID-19.

## Results

**Figure 1:**
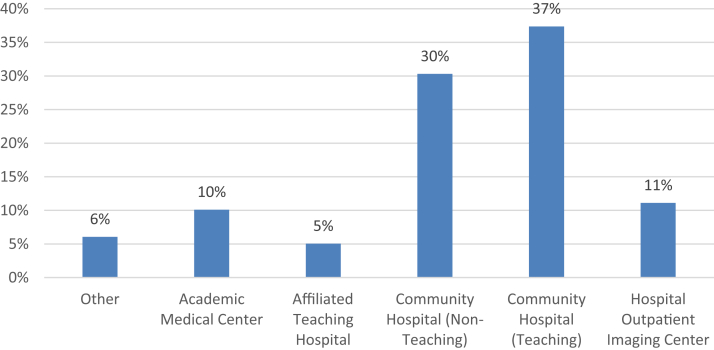
Respondent program by affiliated hospital type (N = 99).

**Figure 2:**
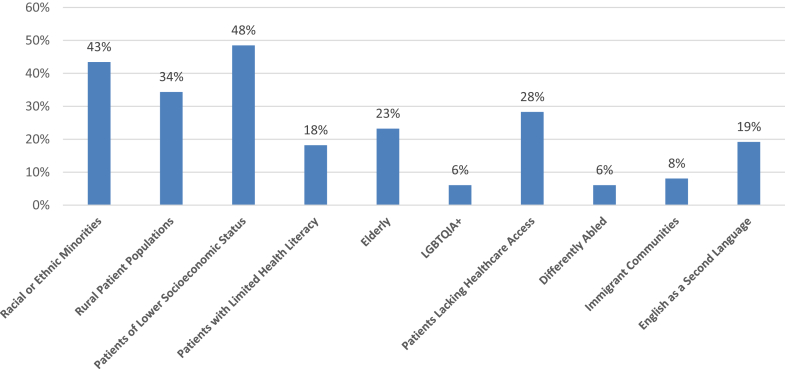
Percent of programs reporting they have specific outreach programs or formal initiatives for the respective populations (N = 99).

In 2021, 99 programs representing 1112 distinct SCOEs completed the survey. Programs represented a national sample with the South, North, Midwest, and Western regions representing 33%, 28%, 25%, and 13% of respondents, respectively. Together, 78% of programs practiced in the community setting, with 67% representing community hospital-affiliated programs, including both teaching and non-teaching sites, and 11% representing hospital outpatient imaging centers. Academic medical centers represented 10% of the respondents (Fig. 1). Programs reported a median of 868 patients (range: 0–7930; SD 1267) screened in 2020. Responding programs noted a wide range of targeted outreach to populations experiencing health disparities (Fig. 2), most often including those of lower socioeconomic status, racial or ethnic minorities, and rural patient populations.

**Figure 3:**
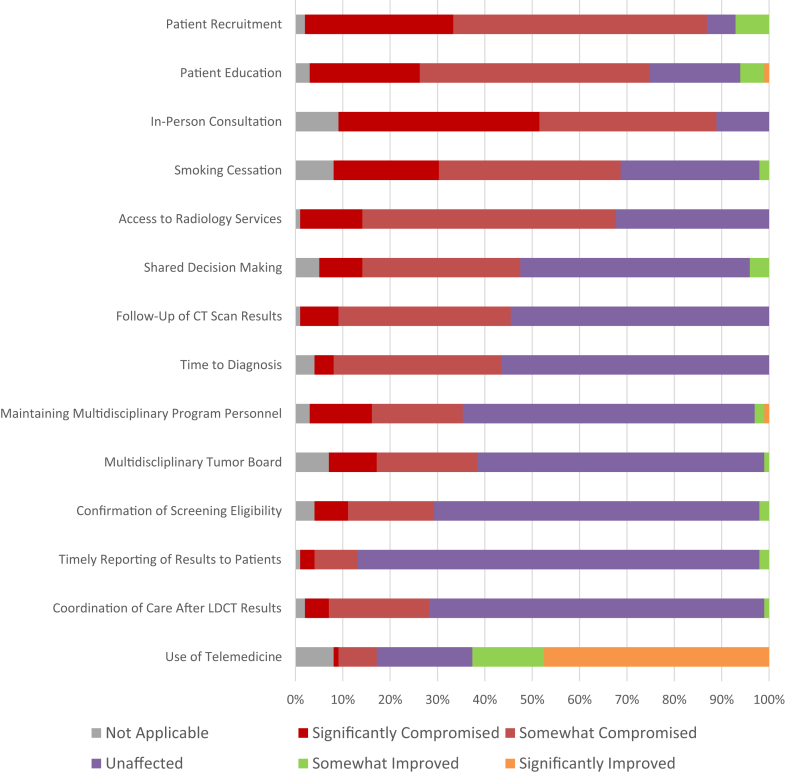
The figure represents the percentage of LCS program components reported as either significantly improved, somewhat improved, unaffected, somewhat compromised, or significantly compromised as a result of the COVID-19 pandemic. COVID-19, coronavirus disease 2019; CT, computed tomography; LCS, lung cancer screening; LDCT, low-dose CT.

Of the respondents, 85% reported a compromise of patient recruitment and 71% reported compromised patient education. Similarly, 79%, 67%, and 60% of the respondents reported compromised in-person consultations, smoking cessation services, and access to radiology services, respectively, due to COVID-19. Infrastructure post-referral remained largely unaffected: confirmation of eligibility (69%), timely reporting of results (85%), care coordination after LCS (71%), follow-up care participation (55%), time to diagnosis (57%), multidisciplinary care (62%), and multidisciplinary tumor board (61%) were reported as unaffected. Nevertheless, 44% of the programs reported compromised follow-up of CT scan results and 39% of the programs reported significantly or moderately compromised time to diagnosis (Fig. 3).

## Discussion

High-quality LCS is a continuum of care from patient education to the LDCT and follow-up care. Previous reports have revealed patient volume decreasing and time to diagnosis increasing amid COVID-19.^7^ Nevertheless, a multi-institutional assessment of how COVID-19 has affected program components has not previously been described. Our work explores LCS from the program perspective, representing LCS Centers of Excellence across the United States, including regions of the South, North, Midwest, and West.

Our cohort represents a diversity of LCS delivery settings including academic centers, community-based centers, and outpatient imaging centers. Reports of LCS have traditionally been from academic programs that have practice delivery models that are different from community-based programs. Though some questions arose about the feasibility and effectiveness of LCS in community-based settings,^10^ Copeland et al.^9^ validated comprehensive LCS in a community setting with early stage lung cancers being detected at an early, more treatable stage. Our study builds on this experience, with 78% of respondents being from community-based programs or imaging centers. Overall, our work reveals resilience in restarting LCS after expert consensus advising against LCS amid COVID-19. For all surveyed programs, the benefits of LCS for patients and institutions outweighed the risks, despite limited resources, potential COVID-19 patient exposure, and fewer personnel.

The uptake of LCS is low nationwide, which is concerning as marginalized groups are even more vulnerable to health disparities.^4^ Our data reveal that only 48% of programs actively recruit individuals from low socioeconomic status populations and only 43% actively recruit from racial or ethnic minorities. Historically, these populations have lower LCS eligibility. Individuals who belong to both groups are likely to have even lower LCS eligibility and are more likely to be missed in screening, leading to worse outcomes.^11^ In addition, individuals living in rural areas have higher incidence of cigarette smoking but are less likely to undergo LCS due to a lack of LCS access.^4^ Among our cohort, only 38% of programs have a targeted recruitment approach for rural populations, whereas a mere 28% are focused on individuals with limited health care access. The survival benefit of LCS is currently only experienced by individuals with the education, access, and priority to screen. This represents a missed opportunity to save lives and decrease the risk of lung cancer, particularly among vulnerable populations that already face health disparities. Our data reveal that LCS can play a crucial role in advancing health equity by focusing education and recruitment on populations facing health disparities.

In this cohort of LCS programs, key components of LCS were adversely affected. COVID-19 had the most impact on patient recruitment and patient education. The unpredictability of COVID-19 surgeries and the availability of personnel to conduct LCS lead to passive promotion, education, and recruitment of screening. In addition, several studies were published in 2020 that affected guidelines for LCS.^3^^,^^4^^,^^6^ Programs may have been reluctant to develop patient outreach while recommendations were still evolving. In-person consultation, smoking cessation, access to radiology services, and shared decision-making are essential components of LCS which were compromised by COVID-19, but less so than patient education and recruitment. Failing to provide smoking cessation services is a missed opportunity, whereas shared decision-making enhances patient follow-up and ensures treatment aligns with their goals. These findings highlight the need for patient-centered tools and systems to support education, smoking cessation, shared decision-making, and access to follow-up care.

The quality of care after referral was well-maintained, despite some areas being compromised. The confirmation of eligibility, follow-up care, time to diagnosis, coordination of care after LDCT results, and maintaining multidisciplinary personnel and tumor board all seemed to be prioritized. This may be due to the commitment of programs once a patient was established within their center. Although recruitment could be a primary limiting factor in LCS uptake once the patients are enrolled, programs may find it easier to continue their follow-up care with the emergence of telehealth.

With in-person visits halted, 79% of programs reported in-person consultation as somewhat or significantly affected by COVID-19, potentially leading to delayed lung cancer diagnosis and treatment, especially for underserved populations.^8^^,^^12^ On a positive note, more than 62% of the programs reported increased telemedicine use for LCS, offering a promising avenue for access. Before the pandemic, less than 1% of health care was conducted through telehealth. Given a substantial amount of funding behind telehealth-based screening is set to expire, advocacy for permanent telehealth funding is crucial.^13^^,^^14^ Though the scan must be at an accredited center, other services can be facilitated through telemedicine. It is important for insurers to access telemedicine paradigms. Nevertheless, further exploration is needed to determine patient acceptance of telemedicine as a mainstay in LCS.

Our study method has limitations. Given that the survey is voluntary, the composition of respondents varies yearly, and participating programs may sway results. In addition, our survey instrument relies on a qualitative assessment of the COVID-19 impact on each LCS program component rather than objective measures. The pandemic-specific questions were also only administered in the annual survey in 2021, which limits our ability to conduct a longitudinal assessment.

In conclusion, our study representing a large national sample size of diverse LCS programs’ experiences amid COVID-19 reveals rapid adaptation to deliver LCS. Nevertheless, patient education and patient recruitment are components of LCS programs that are most likely to be compromised in times of uncertainty and limited resources. Without concerted efforts to educate and recruit all patients at high risk of lung cancer about LCS, screening rates will remain low, especially among patients facing health disparities.

## CRediT Authorship Contribution Statement

**Valeda Yong:** Conceptualization, Formal analysis, Investigation, Methodology, Project administration, Visualization, Roles/Writing - original draft, Writing - review and editing.

**Lynde Lutzow:** Funding acquisition, Conceptualization, Formal analysis, Methodology, Investigation, Project administration, Visualization, Roles/Writing - original draft, Writing - review and editing.

**Andrew Ciupek:** Conceptualization, Data curation, Methodology, Investigation, Project administration, Supervision, Validation, Visualization, Writing - review and editing.

**Angela Criswell:** Conceptualization, Data curation, Methodology, Investigation, Project administration, Writing - review and editing.

**Jennifer C. King:** Conceptualization, Data curation, Methodology, Investigation, Project administration, Supervision, Validation, Visualization, Writing - review and editing.

**Grace X. Ma:** Funding acquisition, Conceptualization, Methodology, Resources, Software, Validation.

**Cherie P. Erkmen:** Funding acquisition, Conceptualization, Methodology, Project administration, Supervision, Validation, Visualization, Writing - review and editing.

## Disclosure

This work was (partially) supported by the National Cancer Institute of 10.13039/100000002National Institutes of Health (NCI/NIH) (TUFCCC/HC Regional Comprehensive Cancer Health Disparity Partnership, Award Number U54 CA221704[5]).
